# Seroprevalence and Risk Factors for Hepatitis C Virus Infection among General Population in Central Region of Yemen

**DOI:** 10.1155/2012/689726

**Published:** 2012-12-18

**Authors:** Rajesh N. Gacche, Sadiq K. Al-Mohani

**Affiliations:** Biotechnology Department, School of Life Sciences, S.R.T.M. University, Vishnupuri, Nanded 431606, India

## Abstract

*Background*. Hepatitis C virus (HCV) represents a major worldwide public health problem. Though several studies from Yemen have provided an estimate of the prevalence of this viral infection, there exist only few studies which reflect the status in the general population. *Aim*. The present study was designed to investigate the prevalence of hepatitis C infection among general population in central region of Yemen. *Methods*. The study population comprised 2,379 apparently healthy subjects who were screened for hepatitis C antibodies (HCV Abs) status using ELISA quantitative technique. Seroprevalence rate of seropositive subjects was calculated and stratified by age, sex, educational level, and monthly income. *Results*. The study showed that out of 2,379 subjects, 31 (1.3%) were HCV Abs positive. Higher prevalence of HCV Abs was found among females, 24 (1.01%), than males, 7 (0.29%). The age specific prevalence rose from 00 (0.00%) in subjects aged ≤14 years to a maximum of 9 (0.38%) in subjects aged ≥55 years. The prevalence of HCV Abs was more prevalent in illiterate subjects and increased with decreasing monthly income. *Conclusion*. It was found that variables including age and educational level were significantly associated with HCV Ab positivity and not associated with gender and monthly income.

## 1. Introduction

Hepatitis C virus has been considered to be one of the most potential pathogens that have hindered the medical community all over the world. Indeed, since its discovery in 1989, hepatitis C virus (HCV) has been recognized as a major cause of chronic liver disease worldwide and due to the surpassing hepatitis B virus [1]. HCV represents a major health problem with approximately 3% of the world population, that is, more than 170 million people infected. While only 20–30% of individuals exposed to HCV recover spontaneously, the remaining 70–80% develop chronic HCV infection. Moreover, 3–11% of those people will develop liver cirrhosis within 20 years, with associated risks of liver failure and hepatocellular carcinoma (HCC) [2]. It is now widely recognized as one of the common aetiological agents for cirrhosis of the liver. It is the leading cause of liver transplantation and the most common chronic blood borne infection in developed countries like the USA [3]. The socioeconomic impact of HCV infection is therefore tremendous, and the burden of the disease is expected to increase around the world as the disease progresses in patients who contracted HCV years ago. Since the discovery of HCV more than 20 years ago, epidemiological studies have described complex patterns of infection concerning not only the worldwide prevalence of this virus but also its clinical presentation and its therapeutic response [2]. Although HCV is endemic worldwide, there is a large degree of geographic variability in its distribution. Countries with the highest reported prevalence rates are located in Africa and Asia; areas with lower prevalence include the industrialized nations [4]. In European region, the prevalence of HCV in the general population varies from 0.4% in Sweden, Germany, and The Netherlands to more than 2-3% in some Mediterranean countries. In Western Pacific region, most countries have prevalence rates from 1% to 2%, some countries have relatively high prevalence rates, including Taiwan (4.4%) and Vietnam (2–2.9%). In Africa region, the prevalence of HCV is even higher in some areas, reaching levels of up to 10% [5]. In the Arab World, the WHO estimates that HCV prevalence is 1–4.6%, with levels higher than 20% in Egypt [5, 6]. Yemen is the second largest heavily populated and the poorest country in Arabian peninsula; the prevalence of HCV in Yemenis was found to be 1.7% among healthy volunteers though it reached 2.7% among blood donors. Such prevalence reached up to 60% in haemodialysis patients [1]. Data on burden of HCV infection in Yemen come primarily from studies on HCV Ab seroprevalence. A number of studies on the epidemiology of HCV infection have been carried out in this country over the last two decades. There are several levels of variability amongst these studies which include the sample size, the methodology for assay of HCV Ab, the age group covered, general population sample versus blood donors, risk-population samples, ethnicity, and geography of the study groups. All these factors have also been shown to influence the prevalence of HCV infection. There are conflicting reports on the prevalence rates of HCV Ab in Yemen. Adequate data on age, sex, educational level, and monthly income specific prevalence is also lacking. Therefore the present study was undertaken to estimate the age, sex, educational level, and monthly income specific prevalence of HCV Ab in general population in central region of Yemen. Ibb governorate is located in the central part of the country with an area of 5,344 square kilometers and a population of approximately 1757028 inhabitants making it the most densely populated governorate in Yemen outside of Sana'a city (capital city of Yemen). It is known as “The Green City” [7] so it receives each year many of tourists from different cities in Yemen and from outside of Yemen. Although Ibb is the most densely populated governorate in Yemen outside of Sana'a city and it has the largest Yemeni expatriates abroad distributed in USA, Saudi Arabia, and others Arab countries, the data for prevalence of viral hepatitis in Ibb governorate are rare and inadequate as well as there is no epidemiological study about prevalence of hepatitis C virus infection in Ibb city, so Ibb was selected for this study.

## 2. Methods

Two thousand and three hundred and seventy nine subjects were randomly selected by systematic random sampling of every fifth house in Ibb city from July 2010 to August 2011 with a 100% response rate. Three milliliter blood sample from apparently healthy subjects belonging to all socioeconomic group was collected in EDTA vacutainers. All subjects were screened for hepatitis C virus antibodies by using one step cassette style anti-HCV device as per instructions from the manufacturer (Rapid Anti-HCV Test, Intec, China). Positive samples were confirmed by enzyme-linked immunosorbent assay (DRG, HCV antibodies, USA). The subjects were categorized according to their respective sex, age, educational level, monthly income, and correlated with risk factors. All collected data were analyzed using SPSS program.

## 3. Results

### 3.1. Overall and Gender Specific Prevalence

The overall prevalence of HCV Abs among subjects aged 7–83 years was 1.3% (*n* = 2, 379). The prevalence of HCV Abs in males was 7 (0.29%) compared to 24 (1.01%) in females. The difference in prevalence between men and women was not statistically significant (*P* = 0.082) (Table 1). 

### 3.2. Age Specific Prevalence

HCV Abs seroprevalence increased with age, 00 (0.00%) in ≤14 year age group to 9 (0.38%) in ≥55 year age group. The difference was found to be statistically significant (*P* < 0.001) (Table 1). The study showed maximum prevalence of HCV Abs at 3 (6.3%) in males aged ≥55 years and at 6 (10.2%) in females aged ≥55 years. For both genders minimum prevalence was seen in the subjects aged less than 14 years (Table 2).

### 3.3. Educational Level Specific Prevalence

The present study showed highest seroprevalence of HCV Abs among illiterate subjects 14 (0.59%) and lowest in postgraduate subjects 00 (0.00%). The difference was found to be statistically significant (*P* > 0.001) (Table 1).

### 3.4. Monthly Income Specific Prevalence

The study showed maximum prevalence of HCV Abs at 21 (0.88%) in subjects with monthly income less than Yrs 20,000 (US$ 93) and minimum prevalence was seen in subjects with monthly income more than Yrs 60,000 (more than US$ 279). We could not establish any significant association between monthly income in relation to HCV Abs positivity (data statistically insignificant).

## 4. Discussion

### 4.1. Overall Prevalence

Hepatitis C is often diagnosed accidentally and, unfortunately, remains heavily underdiagnosed. It is estimated that only 30–50% of individuals infected with HCV are aware of their disease and can take advantage of treatment options and avoid the risk of further transmission of the virus. Serologic tests are sufficient when chronic hepatitis C is expected, with a sensitivity of more than 99% in the 3rd generation assays. Positive serologic results require HCV RNA measurement in order to differentiate between chronic hepatitis C and resolved HCV infection from the past. Qualitative and quantitative HCV RNA assays are available and now being widely replaced by real-time PCR-based assays that can detect HCV RNA over a very wide range, from low levels of approximately 10 IU/mL up to 10 million IU/mL [8]. Hepatitis C is found throughout the world, and it has no seasonal distribution. The reported prevalence of carrier in different population varies widely from 0.3% in the advanced countries to 28% in the developing nations [9]. Transmission of HCV was strongly associated with intravenous and percutaneous drug users (IDUs). The hepatitis C European network for cooperative research group reported a prevalence of hepatitis C of 80% among intravenous drug users (IVDU), 40% in Thailand, and up to 74% in Australia [1]. Risk factors for HCV transmission differ between developed and developing countries. Most developed countries have accumulated evidence that the predominant source of new HCV infections within their borders over the past few decades is injection drug use [10]. In the developing world, unsafe therapeutic injections and transfusions are likely to be the major modes of transmission, especially in countries where age-specific seroprevalence rates suggest ongoing increased risk of HCV infection [11]. Hepatitis C virus prevalence rates are estimated to range between 5.5% in Africa, 4.6% in the Eastern Mediterranean region, 4% in the Western Pacific region, 2% in South East Asia, 1.7% in the United States of America (USA), and 1% in Europe [12]. There has been very high prevalence rate of HCV reported in Egypt in the past 28% [9]. The study showed 1.3% seroprevalence of HCV Ab among general population in central region of Yemen (Ibb city). The frequency of HCV Ab seropositivity was found to be higher than that reported in southern part of Yemen (Aden city) 0.6% and lower than northern part of Yemen (Sana'a city) 2.3% and southern islands (Socotra island) 5.1% [13]. Such differences in prevalence rates may be explained by differences in health resources and educational levels in different regions in Yemen or due to methodological differences between studies.

### 4.2. Sex Specific Prevalence

The prevalence of HCV in both genders is controversial. While some studies showed higher HCV incidence among men, other population based surveys showed slightly higher rates in women than in men. However, additional epidemiological studies are needed to confirm that the risk of HCV transmission is greater in men [14]. In this study, seroprevalence was higher in females, 24 (1.01%). Some other studies have shown similar results [15–17].

### 4.3. Age Specific Prevalence

The present study revealed significant trend of HCV seropositivity with relation to age. The positivity for HCV Abs increased with age. Some other studies have shown similar results [15–18]. Higher prevalence in older age groups may be attributed to their exposure status to various HCV risk factors which was quite evident from the life style and history of the individuals sampled for this study.

### 4.4. Educational Level Specific Prevalence

The present study showed significant association between educational level and HCV Ab seroprevalence (*P* < 0.001). High prevalence of HCV in illiterate people may be attributed to many factors, such as paucity of health education programs about chronic viral hepatitis in educational system and inaccurate estimates of the burden of disease in study area. The study showed maximum prevalence of HCV Abs among illiterate people aged ≥55 years.

### 4.5. Monthly Income Specific Prevalence

The prevalence of HCV Abs increased with decreasing monthly income. Economic status of people affects health status in the country. Yemen is the second largest heavily populated and the poorest country in Arabian peninsula [1]. 27% of people live under the food poverty line, and 42% are under the national income poverty line. In Yemen, poverty is more of a rural than urban phenomena [19]. Monthly income of the individual plays an important role in determining the level of health status. As is well known poverty is a key factor in the spread of common diseases. Yemeni people are the poorest in the Arab region and least developed nation in the world.

## 5. Conclusion

In conclusion, the results of the present study hint at the importance of presenting integrated information and educational programs among these target groups particularly the less literate people for preventing and controlling HCV transmission. Further, future surveillance studies warranting investigations for this viral infection in a broader population would enable us to determine strategies for combating the threats caused by hepatitis C virus. There are some geographic areas in the country that may be at high risk. Control strategies should take these differences into account.

## Figures and Tables

**Table 1:** Relationship between HCV Ab seropositivity and selected variables among general population in central region of Yemen.

Risk factors	HCV Ab (positive)		*P* value
	No	%	
Sex			
Male	7	0.29	0.082
Female	24	1.01	
Age group (years)			
≤14	00	00	<0.001
15–22	5	0.21	
23–30	6	0.25	
31–38	4	0.17	
39–46	1	0.04	
47–54	6	0.25	
≥55	9	0.38	
Educational level			
Cannot read or write	14	0.59%	<0.001
Can read and write	4	0.17%	
Basic education	1	0.04%	
Secondary education	8	0.34%	
Undergraduate education	4	0.17%	
Postgraduate education	00	00	
Monthly income (Yrs)			
≤20.000	21	0.88	0.265
20,000–40.000	9	0.38	
>40,000–60.000	1	0.04	
>60,000	00	00	

**Table 2:** HCV Abs positivity in association with sex and age.

Age group (years)	Male		Female	
	*n/N *	%*	*n/N *	%
≤14	00/139	00	00/115	00
15–22	00/352	00	5/532	0.94
23–30	1/171	0.59	5/434	1.15
31–38	3/73	4.1	1/197	0.51
39–46	00/45	00	1/131	0.76
47–54	00/27	00	6/56	10.7
≥55	3/48	6.3	6/59	10.2

*n*: number of infected persons in each mentioned age group. *N*: total number of age group. %*: the percentage of HCV infection was calculated in relation to total number of persons in each age group not to total number of persons in the study sample.
